# Provider-agency fit in addiction services organizations: implications for evidence-based practice implementation

**DOI:** 10.1186/1940-0640-10-S1-A52

**Published:** 2015-02-20

**Authors:** Alex T Ramsey, Carissa van den Berk-Clark, David Patterson

**Affiliations:** 1 School of Social Work, Washington University in St. Louis, St. Louis, MO, 63130, USA; 2 Department of Psychiatry, Washington University in St. Louis, St. Louis, MO, 63110, USA

## Background

Addiction services organizations have been slow to adopt and implement evidence-based practices (EBPs) for substance abuse and dependence. This is due in part to poor worker morale and organizational climates that are not conducive to successful learning and integration of these practices [1]. Person-organization fit theory suggests that alignment, or fit, between provider- and agency-level characteristics involving the implementation of EBPs may influence provider morale and organizational learning climate and, thus, implementation success [2]. The current study hypothesized that discrepancies, or lack of fit, between provider- and agency-level contextual factors would negatively predict provider morale and organizational learning climate, outcomes shown to be associated with successful EBP implementation.

## Methods

Direct service providers (N = 120) from four addiction services organizations in a large Midwestern city responded to a survey assessing provider morale, organizational learning climate, agency expectations for EBP use, agency resources for EBP use, and provider attitudes towards EBP use. Difference scores between provider- and agency-level factors were computed to model provider-agency fit. Linear regression models were accounted for in all analyses, but were determined to be insufficiently sensitive in modeling the curvilinear (inverted U-shaped) relationships expected in this study. Therefore, quadratic regression analyses were conducted to more adequately model the level of the dependent variables across the entire “fit continuum.”

## Results

**Figure 1:**
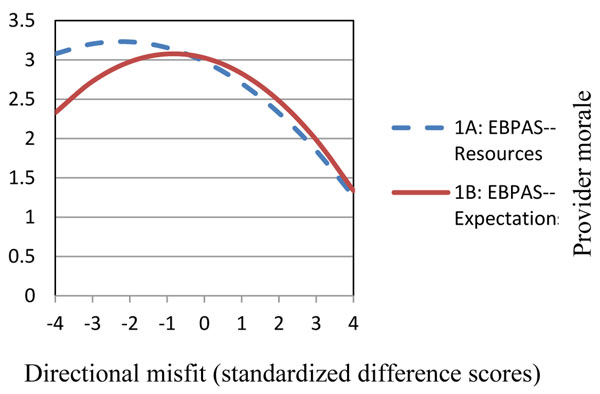
Provider-agency misfit on provider morale.

**Figure 2:**
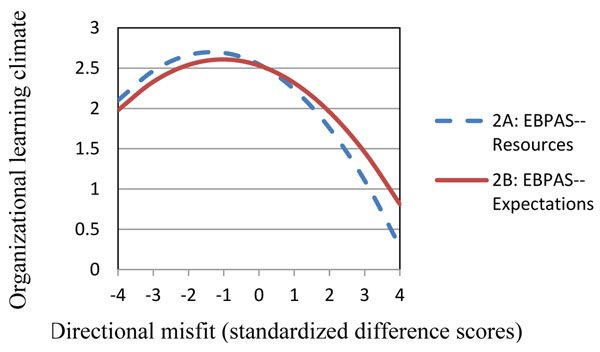
Provider-agency misfit on organizational learning climate. *Note.* A score of zero indicates a perfect fit between provider attitudes and agency characteristics. Positive scores reflect provider attitudes being more favorable towards EBPs than agency characteristics. Negative scores reflect agency characteristics being more favorable towards EBPs than provider attitudes.

Misfit between agency expectations and provider attitudes and between agency resources and provider attitudes were associated with poorer provider morale and weaker organizational learning climate. For all hypotheses, the curvilinear model of provider-agency misfit significantly predicted provider morale and organizational learning climate (Figures 1 and 2). Morale and climate outcomes were most negative when addiction service providers had positive EBP attitudes, but perceived that their respective agency's expectations and resources were not supportive of EBP use.

## Conclusions

This research benefits from a strong theoretical framework, consistent findings, and significant practical implications for substance abuse treatment agencies. Provider morale and organizational learning climate are important indicators of successful EBP implementation. Comprehensive attempts to strengthen these outcomes must consider both provider- and agency-level characteristics regarding EBP use. Managers and supervisors should consider conducting periodical self-assessments of their agency’s cultural predispositions toward EBP implementation (e.g., communicated expectations, supportive resources, technical assistance) and addiction service providers’ openness, abilities, and general attitudes towards using EBPs. Organizational efforts to more closely align provider attitudes and agency priorities will likely constitute a key strategy in fostering the implementation of EBPs in addiction services organizations.
